# The effect of crocin and losartan on *TGF-β* gene expression and histopathology of kidney tissue in a rat model of diabetic nephropathy 

**DOI:** 10.22038/AJP.2022.21414

**Published:** 2023

**Authors:** Yaser Mohammadi, Mohammad Zangooei, Mahmoud Zardast, Morteza Mamashli, Azam Rezaei Farimani

**Affiliations:** 1 *Qaen School of Nursing and Midwifery, Birjand University of Medical Sciences, Birjand, Iran*; 2 *Department of Clinical Biochemistry, School of Medicine, Birjand University of Medical Sciences, Birjand, Iran*; 3 *Medical Toxicology and Drug Abuse Research Center, Department of Pathology, School of Medicine, Birjand University of Medical Sciences, Birjand, Iran *; 4 *Cardiovascular Diseases Research Center, Birjand University of Medical Sciences, Birjand, Iran*

**Keywords:** Diabetic nephropathy, Crocin, Losartan, Kidney, TGF-β

## Abstract

**Objective:**

Diabetic nephropathy is one of the most common microvascular complications of diabetes mellitus that finally leads to complete loss of kidney function. Therefore, this study aimed to evaluate the effect of crocin and losartan on *TGF-β* gene expression and histopathology of kidney tissue in a rat model of diabetic nephropathy.

**Materials and Methods:**

Forty male Wistar rats were randomly divided into five groups (n=8): Untreated control, Diabetic (D), D + crocin, D + losartan, and D + losartan + crocin. Induction of diabetes was performed using streptozotocin (50 mg/kg/ Intraperitoneal injection). At the end of the eight-week period, the rats were sacrificed. Spectrophotometry measured serum glucose, urea, creatinine, and uric acid levels. Microalbumin and creatinine levels were measured in 24-hour urine. Real-time PCR was used to determine the relative expression of the *TGF-β* gene in kidney tissue. Renal tissue histopathology was also examined.

**Results:**

The results showed that hyperglycemia increased biochemical factors associated with diabetes, *TGF-β* gene expression, and kidney damage. Separate treatment with crocin and losartan led to a decrease in renal function factors and *TGF-β* gene expression and improved kidney damage.

**Conclusion:**

Our results showed that crocin could improve kidney function in diabetic conditions. In addition, we showed that crocin increases the effectiveness of losartan. Consequently, we suggest that crocin in combination with chemical drugs can be a potential therapeutic agent for diabetes and its complications. Nonetheless, human studies are needed to make firm findings.

## Introduction

Diabetes mellitus (DM) is a metabolic disease with a marked increase in blood glucose due to impaired insulin secretion or impaired insulin function. Prolonged hyperglycemia with metabolic and hemodynamic imbalance causes oxidative stress and inflammation. It damages multiple body organs, particularly the blood vessels, heart, eyes, nerves, and kidneys, resulting in dysfunction and organ failure. (Abou-Hany et al., 2018).

Diabetic nephropathy (DN) is a fatal diabetic complication expected to rise rapidly due to the growing prevalence of diabetes and the aging population (Hoogeveen, 2022). DN is also the most common cause of chronic renal failure which can progress to kidney failure (Shi et al., 2018). Albuminuria, basement membrane thickening, mesangial matrix enlargement, and extracellular matrix accumulation are the first signs of the disease, followed by glomerulosclerosis and tubular fibrosis (Piccoli et al., 2015). The specific etiology of DN is unknown at this time. Scientists have proposed several mechanisms to explain the pathogenesis of DN, including hyperglycemia, activation of the renin-angiotensin-aldosterone system (RAAS), cytokines, and various growth factors (Ni et al., 2015; Piccoli et al., 2015). Hyperglycemia leads to an increase in transforming growth factor β (*TGF-β*), which is involved in causing glomerulosclerosis and interstitial fibrosis in DN (Mullen and Wrana, 2017). *TGF-β* causes polymerization and an unusual increase in the extracellular matrix by inhibiting the expression of proteoglycans and inducing more collagen synthesis of types I and IV (Isaka, 2018). Because RAAS activation stimulates the generation of *TGF-β* expression, RAAS inhibitors have anti-fibrogenic and anti-inflammatory effects (Faten and Mohammad, 2021; Zhang et al., 2017). So, blocking the RAAS has been the main goal of treating Type 1 diabetes (T1D) patients with microalbuminuria. In the therapeutic management of chronic kidney disease (CKD), it is now well known that medication with angiotensin I-converting enzyme inhibitors or angiotensin II (Ang II) receptor blockers is strategically essential. Losartan, a selective antagonist of the Ang II type 1 (AT1) receptor, has renoprotective effects by inhibiting renal oxidative stress, inflammation, and fibrosis (Teles et al., 2009). Because of the adverse side effects of chemical medications, researchers have focused on natural herbal therapies.

Crocin, a carotenoid that provides saffron’s color, was shown to improve insulin sensitivity and lowere serum glucose levels (Shirali et al., 2013). Crocin also has antioxidant and anti-inflammatory effects (Hussain et al., 2021). This study aimed to evaluate the impact of crocin and losartan on *TGF-β* gene expression and histopathology of kidney tissue in rats with DN.

## Materials and Methods

**Experimental animals**

**Figure 1:**
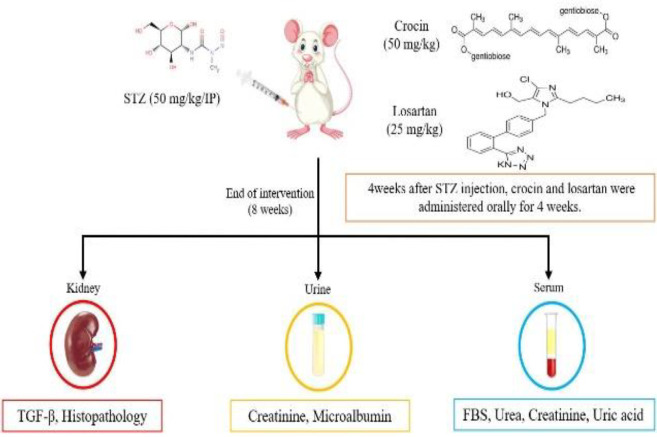
TIMELINE: indicates the study protocol

We used 40 healthy, 3-month-old male Wistar rats (mean weight 200-250 g) purchased from the Experimental Research Center of Birjand University of Medical Sciences, Birjand, Iran. The animals were kept at a constant temperature of 24±2°C and relative humidity of 55%, with a 12-hour light/dark cycle. The animals had adequate access to water and standard rodent food during the study (Figure 1 shows the study protocol in brief).

**Diabetes induction**

Streptozotocin (STZ) powder was purchased from Sigma Aldrich and dissolved in 0.1 mM citrate buffer at pH 4.5. Diabetes was induced experimentally in 32 rats by injecting a single dose of 50 mg/kg freshly prepared STZ intraperitoneally. Rats were given a 15% glucose solution for 24 hr to avoid hypoglycemia. Fasting blood glucose (FBG) was measured from the tail vein after 72 hr by a standard glucometer Accu-Check (Roche, Germany). Rats with FBG >250 mg/dl were considered diabetic. After four weeks of diabetes induction, rats were divided into five groups (n=8) using a randomized blocking procedure: 1) U= Untreated control (Healthy, without treatment and induction of diabetes); 2) D= diabetic control (diabetic rats without treatment); 3) DC= D+ crocin (diabetic rats treated with crocin); 4) DL= D+ losartan (diabetic rats treated with losartan); and 5) DLC= D+ losartan+ crocin (diabetic rats treated with losartan and crocin).

**Treatment**

Crocin powder was purchased from Sigma Aldrich (USA) and losartan from ACTOVERCO (Iran) and dissolved in normal saline. The intervention was started four weeks after induction of diabetes (the 28th day of the study). Crocin (50 mg/kg) and losartan (25 mg/kg) were gavaged daily for four weeks (7 days a week). Losartan and crocin doses were chosen based on previously reported doses in animal research (Alotaibi et al., 2019; Anbar et al., 2016; Ghorbanzadeh et al., 2016).

**Sample collection**

At the end of the experiment, 24-hr urine was collected using metabolic cages to measure microalbumin and creatinine (Cr) and stored in a -20°C freezer. The rats were anesthetized intraperitoneally with ketamine (50 mg/kg) and xylazine (20 mg/kg). Blood samples were drawn from the inferior vena cava. Serum was separated by centrifuging blood tubes at 3000 rpm for 10 min and then frozen at -20°C to measure FBG, urea, Cr, and uric acid (UA). Following kidney isolation, rinsing, and weighing, one was frozen in liquid nitrogen and stored at -80°C, while the other was placed in 10% formalin for histopathological examination.

**Biochemical parameters analyzing**

FBG, urea, Cr, UA, and microalbumin biochemical parameters were measured using standard kits (produced by Pars Azmun, Iran) and an automatic analyzer (Prestige 24i, Japan).

**Estimation of kidney weight/body weight (%)**

At the end of the study, on the 56th day, the left and right kidneys were segregated, the renal fascia was removed, and the kidneys were each weighed separately. The following formula was used to determine the total kidney weight as a percentage of body weight (Kaur et al., 2015).

calculation=left kidney weight g+Right kidney weight (g)Body weight (g)×100

**Total RNA extraction and real-time PCR**

Frozen kidney tissues (50 mg) were homogenized in liquid nitrogen with a pestle. According to the manufacturer's instructions, the total RNA was extracted using the Kiazol Reagent (KIAZIST, Iran). The quality and integrity of isolated RNA were assessed using a denaturing (formaldehyde) 1% agarose gel electrophoresis. The RNA concentration and purity were determined using a NanoDrop UV spectrophotometer (Biotech-America). According to the manufacturer's protocol, the extracted RNA was converted to cDNA using a reverse transcriptase reaction with a cDNA synthesis kit (Pars Tous, Iran). A SYBR green-based real-time PCR assay (Applied Biosystems, USA) was used to measure *TGF-β* gene expression. The amplification protocol included one cycle at 95°C for 10 min, followed by 40 cycles at 95°C for 15 sec, 65°C for 30 sec, and 72°C for 30 sec. β-actin gene was used as an internal control gene, and all the RT-PCR reactions were run in duplicate. The 2^-ΔΔCT^ method was applied to calculate the relative abundance of mRNA transcripts. The sequence of primers is reported in Table 1. 

**Table 1:** Primer sequences used for real-time PCR

**Gene**	**Primer sequence**
**β-Actin**	*Forward*: 5′‐CGCGAGTACAACCTTCTTGC‐3′ *Reverse*: 5′GTCTACAACATGATCTGGGTCA3′
**TGF-β**	*Forward:* 5′‐GCAACAATTCCTGGCGTTAC‐3′ *Reverse*:5′‐GTATTCCGTCTCCTTGGTTCAG‐3ʹ

**Histopathology of kidney tissue**

Kidney tissues were fixed in 10% formalin. Tissue passage, paraffin block preparation, and 5-micron section preparation were done. After staining with Hematoxylin-Eosin (H&E), the slides were examined under a microscope (Olympus-BX41, Japan). Mesangioproliferative glomerulopathy, tubular cell glycogen storage, tubular cell necrosis and desquamation, tubulointerstitial congestion, and tubulointerstitial inflammation were assessed in tissue sections to determine the degree of kidney damage by an expert pathologist. According to the degree of various injuries, renal morphological changes were scored as follows: 0 (none), 1 (≤10%), 2 (11–25%), 3 (26–45%), 4 (46–75%), and 5 (≥76%) (Yang et al., 2019). 

**Statistical analyses**

All data from the experimental groups are presented as means±SEM. The Shapiro-Wilk test was used to test the normality hypothesis. Analysis of variance and appropriate post hoc tests were used to compare the groups. A p-value <0.05 was considered to be statistically significant. The analysis was performed using IBM SPSS Statistics 16.0 software (SPSS, Inc., Chicago, IL).

## Results

**Effect of crocin and losartan on FBG**

**Figure 2:**
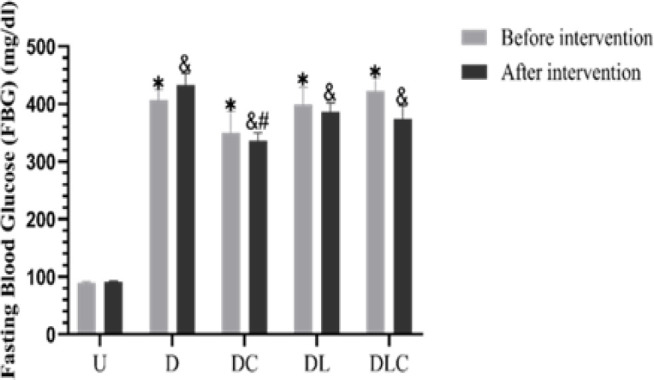
Effect of crocin and losartan on FBG. Data is shown as mean±SEM, N=8; Tukey’s post hoc test was used to compare groups. U (untreated control), D (Diabetic), DC (Diabetic treated with crocin), DL (Diabetic treated with losartan), and DLC (Diabetic treated with losartan and crocin). Before intervention: *p<0.05 significant difference compared with the group U. After intervention: & p<0.05 significant difference compared with the group U, and #p<0.05 significant difference compared with the group D.

FBG increased significantly (p=0.001) in all diabetic groups before the intervention (on the 28th day) compared to the untreated control group (Figure 2). The results showed that four weeks after the intervention (56th day), FBG decreased in the treated groups compared to the diabetic group. In the DC compared to the D group, these differences were statistically significant (p=0.001). In addition, our results showed that FBG was lower in the DLC group than DL group (non-significantly). 

**Effect of crocin and losartan on body weight and kidney/body weight %**

**Figure 3:**
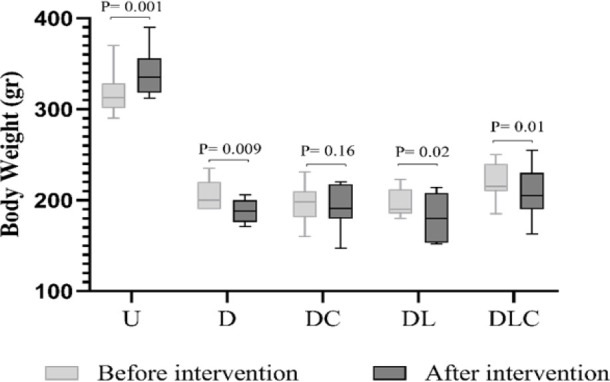
Effect of crocin and losartan on body weight. N=8, Paired T-test was used to compare groups. P values show the statistical differences between the groups. U (untreated control), D (Diabetic), DC (Diabetic treated with crocin), DL (Diabetic treated with losartan), and DLC (Diabetic treated with losartan and crocin).

Figure 3 depicts the changes in body weight before (on the 28th day) and after the intervention (on the 56th day). Before the intervention: the body weight of all diabetic rats was significantly lower compared to the U control group (p=0.001). But after the intervention: the body weight of treated rats increased compared to the D group (non-significantly).

As can be seen, DLC group experienced the greatest weight gain compared to other treated groups (non-significantly). Table 2 shows the average kidney/body weight %. The results showed that the kidney/body weight % in the D group increased significantly compared to the U group (p=0.001). The mean of this index decreased in the treated groups compared to the D group (non-significant). Also, this index decreased more in DLC group compared to DL group, but these changes were not statistically significant.

**Effect of crocin and losartan on serum levels urea, Cr, and UA**

Table 2 shows the serum concentrations of urea, Cr, and UA. The present study results show that serum urea, Cr, and UA levels increased remarkably compared to the U group. Four weeks after treatment, serum levels of urea, Cr, and UA decreased in the DC and DL groups compared to the D group (non-significant). When the DLC group was compared with the DL group, serum urea, Cr, and UA levels slightly improved (non-significant).

**Effect of crocin and losartan on urinary levels of Cr and microalbumin**

Table 2 shows the concentration of urinary Cr and microalbumin. The results showed that the urinary Cr level was significantly increased in the D group compared to the U group (p=0.001). Also, urine Cr levels reduced significantly in the DC and DLC groups compared to the D group (p=0.001 and p=0.01, respectively). Urine Cr levels reduced in the DLC group compared with the DL group (non-significant).

In addition, the amount of urine microalbumin in the D group increased significantly compared to the U group (p=0.001). But, the urine level of microalbumin in the diabetic groups treated with DC, DL, and DLC decreased significantly compared to the D group (p=0.001). Urine microalbumin decreased in the DLC group compared to the DL group but the difference was not statistically significant. 

**Effect of crocin and losartan on** ***TGF-β*** **gene expression**

**Figure 4:**
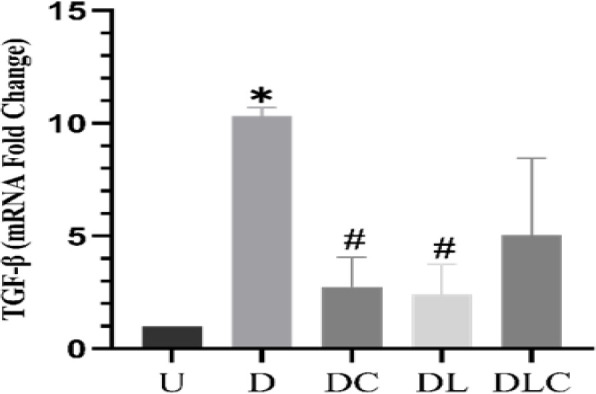
Effect of crocin and losartan on *TGF-β* gene expression in kidney tissue. Data is shown as mean±SEM, N=8, U (untreated control), D (Diabetic), DC (Diabetic treated with crocin), DL (Diabetic treated with losartan), and DLC (Diabetic treated with losartan and crocin). *p<0.05 significant difference compared with the group U, #p<0.05 significant difference compared with the group D.

Figure 4 shows the expression of the *TGF-β* gene in kidney tissue. Our study showed that the expression of the *TGF-β* gene in the D group increased significantly compared to the U (p=0.003). DC and DL significantly reduced *TGF-β* expression in the compared to the D group (p=0.02).

**Table 2:** Effect of crocin and losartan on serum and urinary levels of biochemical parameters, and kidney/body weight ratio

**Variable**	**Groups**				
	**U**	**D**	**DC**	**DL**	**DLC**
**Serum urea (mg/dl)**	39.37±1.49	143±11.5^*^	100.25±5.38^*#^	138.57±9.22^*^	114.71±4.44^*^
**Serum Cr (mg/dl)**	0.68±0.06	0.85±0.02	0.77±0.07	0.82±0.01	0.8±0.03
**Serum UA (mg/dl)**	0.61±0.08	1.37±0.27^*^	0.82±0.05	1.31±0.12^*^	1.11±0.09
**Urinary Cr (mg/dl)**	10.46±0.6	4.2±0.26^*^	8.21±0.64^*#^	4.87±0.5^*^	6.71±0.26^*#^
**Microalbumin (mg/dl)**	1.09±0.33	22.86±3.65^*^	9.52±1.57^*#^	13.71±2.78^*#^	10.15±1.06^*#^
**kidney/body weight % **	0.69±0.02	1.31±0.09^*^	1.28±0.05^*^	1.30±0.03^*^	1.23±0.04^*^

Data is mean±SEM, N=8, U (untreated control), D (Diabetic), DC (Diabetic treated with Crocin), DL (Diabetic treated with Losartan), DLC (Diabetic treated with Losartan and Crocin). *p<0.05 significant difference compared with group U, #p<0.05 significant difference compared with group D.

**Effect of crocin and losartan on** **histopathology of kidney tissue**

The histological abnormalities of kidney tissue are shown in Table 3. Mesangial proliferative glomerulopathy, tubular cell glycogen storage, tubular cell necrosis and desquamation, tubulointerstitial congestion, and tubulointerstitial inflammation increased significantly in the D group compared to the U group (p= 0.001). 

In the treated diabetic groups, DC, DL, and DLC could significantly reduce mesangial proliferative glomerulopathy, tubular cell glycogen storage, tubular cell necrosis and desquamation tubulointerstitial congestion, and tubulointerstitial inflammation kidney damage compared to the D group (p=0.001).

**Figure 5:**
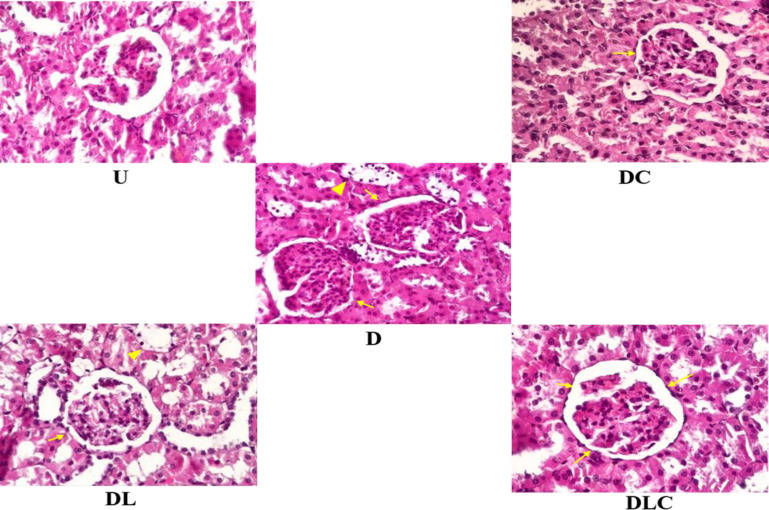
Effect of crocin and losartan on histopathology of kidney tissue. The yellow arrow indicates glomerulopathy, and the yellow triangles indicate tubular cell glycogen storage (Tubulopathy). 400x magnification, H&E coloring. U (untreated control) group; glomeruli and tubules are normal. D (Diabetic) group; Glomeruli with many cells, the tubules by relatively high glycogen stores (tubulopathy). DC (Diabetic treated with crocin) group, glomerulopathy close to the untreated control group, without tubulopathy. DL (Diabetic treated with losartan); glomerulopathy, and tubulopathy are much less. DLC (Diabetic treated with losartan and crocin) group; glomerulopathy and tubulopathy are much less than others.

In addition, histopathological results of renal tissue show that DLC improves kidney damage more favorably than DC and DL. The histology of kidney tissue in the research groups is shown in Figure 5. 

## Discussion

DN is one of the most common microvascular complications of diabetes mellitus. DN is a metabolic disorder caused by chronic hyperglycemia that causes kidney dysfunction (Natesan and Kim, 2021). Furthermore, DN is one of the primary causes of end-stage renal disease (ESRD) and it significantly impacts patient mortality and quality of life (El-Fawal et al., 2018; Natesan and Kim, 2021). Microalbuminuria and a decrease in glomerular filtration rate (GFR) are two symptoms of DN (El-Fawal et al., 2018). Our results showed that in the DC, DL and DLC groups compared to the D group, serum levels of FBG, urea, Cr, UA, microalbuminuria decreased and body weight increased. Also, in the treated groups compared to the D group, the expression of TGF-β decreased and kidney damage improved. Additionally, our findings demonstrated that losartan was more efficient in modifying biochemical parameters and reducing renal tissue damage when combined with crocin. 

**Table 3:** Effect of crocin and losartan on histopathology of kidney tissue

**Variable %**	**Groups**				
	**U**	**D**	**DC**	**DL**	**DLC**
**Mesangioproliferative glomerulopathy**	0.0	58.32±3.63^*#^	36.45±3.5^*#^	33.32±3.63^*#^	29.75±2.48^*#^
**Tubular cell glycogen storage**	3.11±1.51	85.74±4.71^*#^	41.66±3.85^*#^	52.38±5.37^*#^	36.9±3.08^*#^
**Tubular cell necrosis & desquamation**	1.09±1.03	38.09±4.4^*#^	27.07±1.35^*#^	28.55±1.67^*#^	28.55±1.67^*#^
**Tubulointerstitial Congestion**	8.31±2.72	51.18±5.27^*#^	27.07±2.08^*#^	29.75±2.48^*#^	29.75±4^*#^
**Tubulointerstitial Inflammation**	3.11±1.52	39.28±3.51^*#^	22.92±2.07^*#^	28.58±3.57^*#^	23.8±3.36^*#^

Data is mean percentage±SEM, N=8, U (untreated control), D (Diabetic), DC (Diabetic treated with crocin), DL (Diabetic treated with losartan), and DLC (Diabetic treated with losartan and crocin). *p<0.05 significant difference compared with the group U, #p<0.05 significant difference compared with the group D.

Previous studies have shown that after STZ induction, the body weight of diabetic rats begins to decrease (Talebanzadeh et al., 2018). By entering β cells through the glucose transporter 2 (GLUT2), STZ reduces insulin production and results in hyperglycemia (Farshid et al., 2016). Proteolysis and lipolysis may be the leading causes of weight loss in these conditions (Ashrafi et al., 2017). According to the findings of this research, crocin's anti-hyperglycemic impact might prevent body weight loss in diabetic rats. Compared to the diabetic group, Sefidgar et al. found that daily injection of crocin (40 and 60 mg/kg) reduced blood glucose levels and improved body weight. (Sefidgar et al., 2019). Crocin therapy (20 and 30 mg/kg) enhanced the body weight of diabetic rats and lowered blood glucose levels, according to Samarghandian et al. (Samarghandian et al., 2016). Reduced intestinal glucose absorption, improved insulin sensitivity, stimulation of glucose uptake by peripheral cells, increased insulin release from pancreatic β cells, and gluconeogenesis suppression are all proposed reasons for crocin's blood-glucose-lowering effects (Rajaei et al., 2013; Talebanzadeh et al., 2018). Ang II causes oxidative stress and changes insulin signaling, resulting in decreased glucose transport and insulin resistance. Ang II also enhances pancreatic cells' oxidative stress, inflammation, and apoptosis. As a result, inhibiting Ang II signaling is linked to reduced insulin resistance. In this research, losartan, an Ang II receptor antagonist, resulted in lower FBG and body weight increase compared to D group, but the differences were not statistically significant.

Chronic hyperglycemia activates the RAAS system, resulting in an increase in nitric oxide (NO) generation, vasodilation impairment, endothelial damage, glomerular nephropathy, and microalbuminuria (Abou-Hany et al., 2018). Therefore, the kidney loses its ability to filtrate nitrogenous components and increases markers of renal dysfunction (Abou-Hany et al., 2018; El-Fawal et al., 2018). Our results showed that in DC and DL groups, urea serum level decreased. Also, urinary Cr increased significantly in DC and DLC groups. In addition, in all treated groups, microalbumin was significantly reduced compared to the D group. Our results were consistent with a study by El-Fawal et al. (El-Fawal et al., 2018). We showed that crocin might improve renal function markers by decreasing blood glucose. Also, losartan might reduce the damaging effects of Ang II by inhibiting the AT1 receptor and enhancing kidney function (Peruchetti et al., 2021). 

Mesangial and tubular epithelial cell hypertrophy and *TGF-β* production are induced by hyperglycemia and increased Ang II activity (Faten and Mohammad, 2021; Xu et al., 2017). *TGF-β* promotes extracellular matrix thickening, hypertrophy, and enhanced collagen production in mesenchymal cells (Liu et al., 2020). The kidney/body weight %, an indicator of renal hypertrophy, increased in diabetic rats in this study and decreased in the DC group. The diabetic group treated with DC and DL had significantly lower *TGF-β* gene expression in kidney tissue than the D group in our research. Keelo et al. reported that crocin reduced *TGF-β* with its antioxidant properties (Ali Hammood Keelo et al., 2022). Dou et al. found that administering 10 mg/kg of losartan significantly lowered *TGF-β* gene expression in renal tissue by suppressing the *PI3K/Akt/mTOR* pathway (Dou et al., 2019). Based on the findings, we demonstrated that crocin's hypoglycemic impact may have caused a reduction in *TGF-β* gene expression in renal tissue. Losartan may also decrease *TGF-β* gene expression by blocking Ang II.

The histopathology results of renal tissue showed that glomerulopathy, tubular cell necrosis and desquamation, tubulointerstitial congestion, and tubulointerstitial inflammation significantly decreased in the diabetic groups treated with DC, DL and DLC compared with the D group. Also, our results showed that losartan, in combination with crocin, improved kidney damage more effectively than DC and DL.

Yaribeygi et al. reported that crocin improved kidney function and decreased oxidative stress, apoptosis, and inflammation (Yaribeygi et al., 2018). Samadi et al. showed that losartan decreased renal fibrosis by inhibiting Ang II receptor AT1 (Samadi-Noshahr et al., 2021).

It is noteworthy that according to the pharmacological properties of crocin, when this substance is administered orally, it turns into its active metabolite, crocetin. Based on these findings, crocetin probably acts as an essential active metabolite of crocin in the body (Xi and Qian, 2006).

The present study had the following strengths: 1- It is the first study that examined the effects of crocin and losartan on DN. 2- Eight rats were placed in each group due to rat mortality prediction. 3- The intervention started four weeks after induction of diabetes to occur renal damage. 4- We did not lose any rats during the study period. 5- The effect of losartan, crocin, and losartan + crocin was investigated.

The present study had the following limitations: 1- Examining changes in gene expression at the mRNA level; it is unclear whether these changes exist at the protein level. 2- Administering losartan and crocin in a single dose. 3- Shortening the intervention's time due to the possibility of rats mortality. 4- Not investigating the effects of losartan and crocin on healthy rats.

Crocin can improve renal function indicators and histological damages by lowering blood glucose and decreasing *TGF-β*, which causes kidney fibrosis. In addition, we showed that crocin increases the effectiveness of losartan. Consequently, we suggest that crocin in combination with chemical drugs can be a potential therapeutic agent for diabetes and its complications. Nonetheless, human studies are needed to make firm findings.

## Conflicts of interest

The authors have declared that there is no conflict of interest.
